# The efficacy of camel milk and Tarangabin (manna of *Alhagi* *maurorum*( combination therapy on glomerular filtration rate in patients with chronic kidney disease: A randomized controlled trial

**Published:** 2020

**Authors:** Seyed Mousalreza Hoseini, Majid Anushiravani, Mohammad Javad Mojahedi, Maryam Hami, Saeid Zibaee, Hassan Rakhshandeh, Ali Taghipour, Zahra Nikakhtar, Hamid Eshraghi, Amir Parviz Tavassoli

**Affiliations:** 1 *Department of Gastroenterology, School of Medicine, Mashhad University of Medical Sciences, Mashhad, Iran*; 2 *Department of Persian Medicine, School of Persian and Complementary Medicine, Mashhad University of Medical Sciences, Mashhad, Iran*; 3 *Kidney Transplantation Complications Research Center, Ghaem Hospital, School of Medicine, Mashhad University of Medical Sciences, Mashhad, Iran*; 4 *Razi Vaccine and Serum Research Institute, Agricultural Research Education and Extension Organization (AREEO), Mashhad, Iran*; 5 *Pharmacological Research Center of Medicinal Plants, Mashhad University of Medical Sciences, Mashhad, Iran*; 6 *Department of Epidemiology and Biostatistics, School of Health, Mashhad University of Medical Sciences, Mashhad, Iran*

**Keywords:** Camel milk, Tarangabin (manna of Alhagi maurorum), Traditional Persian Medicine, Chronic kidney disease (CKD), Glomerular filtration rate (GFR), Randomized controlled trial

## Abstract

**Objective:**

This study was designed to investigate the effect of camel milk and Tarangabin (manna of *Alhagi maurorum*) combination therapy in addition to conventional treatments in patients with chronic kidney disease (CKD).

**Materials and Methods:**

Forty-four patients of 15 to 70 years old, with CKD due to hypertension or diabetes, and estimated glomerular filtration rate (eGFR) of 15–60 ml/min per 1.73 m^2^, were enrolled in this trial. The patients were randomized to receive either 400 cc of camel milk with 10 cc of Tarangabin syrup orally in two divided daily doses for 3 months plus conventional therapy or conventional therapy alone. The conventional treatment included diabetes medications and angiotensin converting enzyme inhibitors or angiotensin receptor blockers.

**Results:**

The baseline characteristics of patients were similar in the two groups. Serum levels of creatinine (p=0.01), blood levels of urea nitrogen (p=0.0001), triglyceride (p=0.02), and potassium (p=0.05), and diastolic blood pressure (p=0.0001) decreased, while eGFR (p=0.001) improved in intervention group significantly.

**Conclusion:**

It seems that the therapeutic protocol used in this study can improve renal function in patients with CKD through regulating glucose and anti-inflammatory, laxative, and immunostimulatory properties.

## Introduction

Chronic kidney disease (CKD) is a universal health problem with a high economic burden which is proven to be an independent risk factor for cardiovascular disease (CVD) (Eckardt et al., 2013 ▶). CKD is a comprehensive term covering abnormalities of kidney structure or function, being present for more than 3 months (Skorecki et al., 2015 ▶). The most common causes of CKD are diabetes and hypertension. CKD is classified in five stages (1 to 5) according to glomerular filtration rate (GFR) and degree of albuminuria (Goldman and Schafer, 2016 ▶). 

 The mean global CKD prevalence has been estimated to be 13.4 and 10.6%, respectively for all 5 stages and stages 3-5. The prevalence of the disease is 13.7% in 30s and increases with ageing reaching 27.6% in 60s (Hill et al., 2016 ▶). CKD is a serious health problem in low- and middle-income countries (Mills et al., 2015 ▶) where the prevalence rate of end-stage renal disease (ESRD) is growing (Neuen et al., 2017 ▶). 

Some CKD complications include metabolic and endocrine complications, and increased risk for CVD. The complications may occur at any stage of CKD. Slowing down the progression of the disease and reducing its complications are important goals of CKD treatment (KDIGO consortium, 2013 ▶). It is very important to find a safe and useful treatment for patients with CKD. 

Meanwhile, according to the World Health Organization, in many countries, people desire to use complementary and alternative therapies (Al-Rawi and Fetters, 2012 ▶). Base on the principles and clinical teaching of Traditional Persian Medicine (TPM), some therapeutic protocols can be suggested for this group of patients. Evaluating the symptoms and structural changes of CKD, showed that it matches renal weakness (*da’f ol-kolyah*) in TPM. Kidney weakness is characterized by symptoms such as fatigue or lack of energy, swelling in legs or arms, decreased libido, bone or joint pain, muscle soreness, reduced vision, and atrophy in kidneys (*hozal e kolyah*) in TPM. Based on TPM literature, there are various therapeutic protocols for treatment of renal weakness. Camel milk is considered a unique treatment as a kidney tonic in TPM (Arazani, 2009 ▶; Azam Khan, 2008 ▶; Ibn e Sina, 2005 ▶). Also, according to some major *Materia Medica* manuscripts of TPM, camel milk in combination with Tarangabin (manna of *Alhagi maurorum*) is very useful in treatment of kidney weakness (Azam Khan, 2008 ▶; Ibn e Sina, 2005 ▶). 

The efficacy of camel milk on diabetes and its complications was presented in numerous studies. In patients with diabetic nephropathy, the effect of camel milk was observed in terms of reduction of albuminuria, and these patients also showed better blood sugar control (Shori, 2015 ▶; Abdalla, 2014 ▶; Agrawal et al, 2009 ▶). Camel milk reduces oxidative damage in the liver and kidney tissue in animal samples treated with aluminum chloride (Al-Hashem, 2009 ▶). Anti-hyperglycemic, anti-oxidant, and anti-hypertension properties of camel milk were also reported (Salami et al., 2011 ▶). 

The other component of our combined medication is Tarangabin which is a manna exudate produced on the aerial parts of some Alhagi genera such as *A.* *maurorum*. According to TPM scholars, Tarangabin is laxative, detergent, cough reliever, thirst quencher, antipyretic, antiemetic, and body warmer (Ibn al-Baytar, 1992 ▶; Razi, 1971 ▶).

The objective of our research was to evaluate the efficacy of camel milk and Tarangabin on renal function when used in combination with conventional therapy and to compare its potential with the conventional therapy alone.

## Materials and Methods

**Study design**

This randomized controlled trial was designed to investigate the effect of conventional therapy combined with camel milk and Tarangabin in patients with mild to moderate CKD compared with conventional therapy alone and conducted between August 2017 and June 2018. The study was performed at the Clinic of Traditional and Complementary Medicine at Mashhad University of Medical Sciences, Mashhad, Iran. 

 The study protocol was approved by the local ethics committee of Mashhad University of Medical Sciences (Project number: IR.MUMS.REC.1395.437) and patients signed written informed consent before enrollment. The study was registered in the Iranian Registry of Clinical Trials (IRCT) with No. IRCT 2016121531427N1. 

**Study population**

The patients included were those referred by nephrologists to the Clinic of Traditional and Complementary Medicine at Mashhad University of Medical Sciences. The patients were eligible if they were between 15 and 70 years old, had CKD due to hypertension or diabetes with an eGFR of 15-60 ml/min per 1.73 m^2^, hemoglobin A1c (HbA1c) lower than 9, and controlled blood pressure i.e. systolic blood pressure (SBP)≤160 mmHg, and diastolic blood pressure (DBP)≤100 mmHg. Participants with a history of cancer, urinary obstruction, active and febrile infections, use of nephrotoxic drugs, symptomatic cardiovascular disease, liver failure, polycystic kidney, kidney transplants, and pregnancy were not enrolled. The excluded patients were those with acute kidney injury during the study and discontent with any stage of the study. Randomization was carried out using a computer-generated random allocation list. The patients were randomly assigned as 1:1 in the intervention and control groups.

**Intervention**

The intervention group received 400 cc (200 cc in the morning and 200 cc in the evening) of camel milk and 10 cc of Tarangabin syrup 40% daily in two divided doses along the conventional treatments. Camel milk and Tarangabin syrup were consumed separately at the same time. The control group received only conventional medications which were prescribed by the nephrologists. Conventional treatment mainly included diabetes medications for diabetics and angiotensin converting enzyme inhibitors (ACE-I) or angiotensin receptor blockers (ARBs) for hypertensive patients. The patients received their defined treatment for 3 months, and were evaluated for clinical signs and symptoms as well as any drug side effects every month.

**Measurements and outcomes**

Demographic information of patients including age, gender, occupation, and education as well as disease-related information including the underlying illness and their medications were recorded at the first visit. The weight and blood pressure (BP) of patients were measured at each visit. The following biochemical factors were measured before and after treatment at the laboratory of Jahad Daneshgahi of Mashhad: hemoglobin (Hb), serum creatinine, blood urea nitrogen (BUN), uric acid, fasting blood sugar (FBS), HbA1c, sodium (Na), potassium (K), calcium (Ca), phosphorus (P), and lipid profile. 

The primary outcome of the study was the effect of therapeutic protocol on eGFR. The eGFR for each patient was calculated using the four-variable Modifications of Diet in Renal Disease (MDRD) study equation (Levey et al., 1999 ▶). 

**Preparation of Tarangabin syrup and camel milk**

Tarangabin from Torbat-e-Jam city, Khorasan Razavi province, Iran, was purchased from the medicinal plants market and its authenticity was confirmed by a botanist from the Department of Botany Research Center for Plant Sciences, Ferdowsi University of Mashhad, Khorasan, Mashhad, Iran. Tarangabin was manna of *Alhagi maurorum* (Voucher sp. No. E1028-FUMH). High quality Tarangabin was used to prepare 40% syrup at the Pharmacological Research Center of Medicinal Plants of Mashhad School of Medicine as follows: Initially, thorns and chips were removed from Tarangabin; then, Tarangabin was dissolved in distilled water and warmed up to 60°C in bain-marie. The solution was then filtered and centrifuged to remove the suspended particles. Since Tarangabin contains melezitose sugar (47.7%), sucrose (26.44%), and reducible sugar (11.6%), sugar content of Tarangabin syrup 40% was 34.25%. Some sugar was added to this syrup to obtain a concentration of 66.7%. Propyl paraben as little as 0.2% (w/v) was added to the syrup as a preservative (Kulkarni et al., 2012 ▶; Ramezany et al., 2013 ▶). Prepared syrup was poured in a 100-ml glass bottle. It was then placed inside the boiling bain-marie and at the same time, the lid of the glass bottle was closed. This syrup was stored in the refrigerator until the delivery stage. It was strongly recommended to patients that the syrup should be kept in the refrigerator during the period of consumption. Camel milk was collected and prepared for distribution by the Razi Vaccine and Serum Research Institute. The milk was made from Turkmen race camel from Mashhad city, Khorasan Razavi province, Iran, and was approved by veterinarians of Iranian National Scientific Camel Society. The milk was pasteurized at 70°C for 15 min and stored in a refrigerator until delivery. The patients received camel milk every week.

**Sample size**

The sample size was estimated as 22 subjects for each group according to the following formula and was raised to 25 to cover 10% sample loss. Changes of mean GFR and standard deviation were obtained from control group of a similar study (Lin et al., 2008 ▶). The first type error (alpha) was considered 5% along with power of 80% for each group. 

$n=\frac{{\left(z_{1-\frac{\alpha }{2}}+z_{1-\beta }\right)}^{2}(\delta_{1}^{2}+\delta_{2}^{2})}{{\left(\mu_{1}-\mu_{2}\right)}^{2}}$

**Statistical analyses**

The frequency, mean, and standard deviation were used to describe the data. Data normality was examined using the Kolmogorov-Smirnov test. Non-parametric tests were used for abnormal quantitative variables. To analyze the outcomes of the study, if the values were normal, paired t-test was used to compare the values before and after the study in the intervention and control groups. However, if the values were abnormal, Wilcoxon test was used. Mann-Whitney U test was employed for comparing the values between intervention and control groups. All statistical analyses were done using SPSS software program version 20. The significance level was considered 0.05 across all tests. 

## Results

**Participants and baseline characteristics**

**Figure 1:**
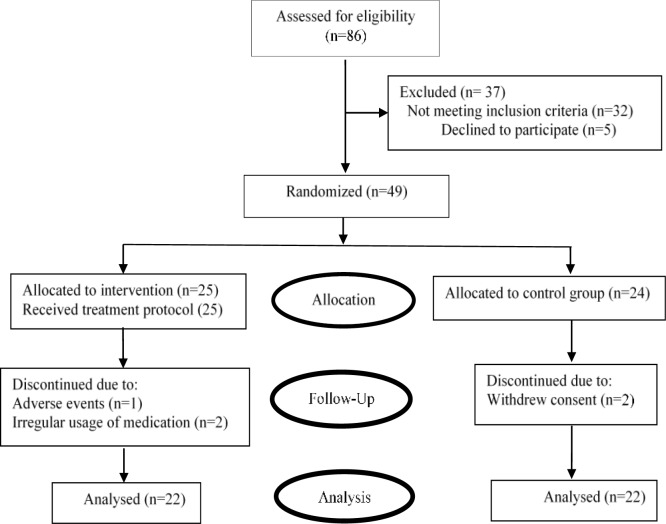
Flow diagram of study

A total of 86 patients were screened for eligibility criteria, of whom 49 were included and randomly assigned into two groups. Two patients in the control group were excluded due to withdrawal from the study and three in the intervention group due to adverse events and irregular usage of medications. A total of 44 patients were analyzed at the end of the trial. The flow diagram of the study is demonstrated in Figure 1.

 The mean age of patients was 56.65±11.76 years old. In this study, men to women ratio was 23/21 (52.3% vs. 47.7%). The underlying diseases were diabetes and hypertension in 23 (52.3%) and 21 (47.7%), respectively of the participants. There was no difference between the two groups in terms of baseline characteristics of patients including demographic, laboratory and underlying diseases at the beginning of the study. There was a significant difference only in gender, so that the proportion of men was higher in the intervention group. The baseline characteristics of patients are presented in Table 1. These characteristics were compared before exclusion of the five above-mentioned participants.

**Table 1:** Homogeneity of baseline characteristics of patients

	**Intervention**	**Control**	**P value**
Demographic characteristics			
Age(Year)*	56.08±10.61	57.62±13.02	0.428
Male**	17(68.0)	9(37.5)	0.034
Body weight (kg)*	70.16±11.19	71.41±11.76	0.703
Systolic blood pressure (mmHg)*	138.40±8.00	142.50±16.41	0.409
Diastolic blood pressure (mmHg)*	90.00±5.20	87.50±18.17	0.261
Underling disease**			
Diabetes	12(48.00)	13(54.1)	0.669
Hypertension	13(52.00)	11(45.9)	
Laboratory characteristics			
Serum Creatinine (mg/dl)*	2.4652±0.74	2.09±0.70	0.367
eGFR( ml /min per 1.73 m^2^)*	29.08±11.01	31.29±11.99	0.504
FBS (mg/dl)*	126.32±45.61	141.20±59.47	0.155
HbA1c %*	6.99±1.27	6.96±1.49	0.912

FBS: Fasting blood sugar and HbA1c: Hemoglobin A1C. * Data are presented as Mean±SD and analyzed by T test if had normal distribution or Mann-Whitney U test if had abnormal distribution. **Data are presented as number (%) analyzed by Mann-Whitney U test

**Effect of combination therapy of camel milk and Tarangabin on renal function**

**Figure 2:**
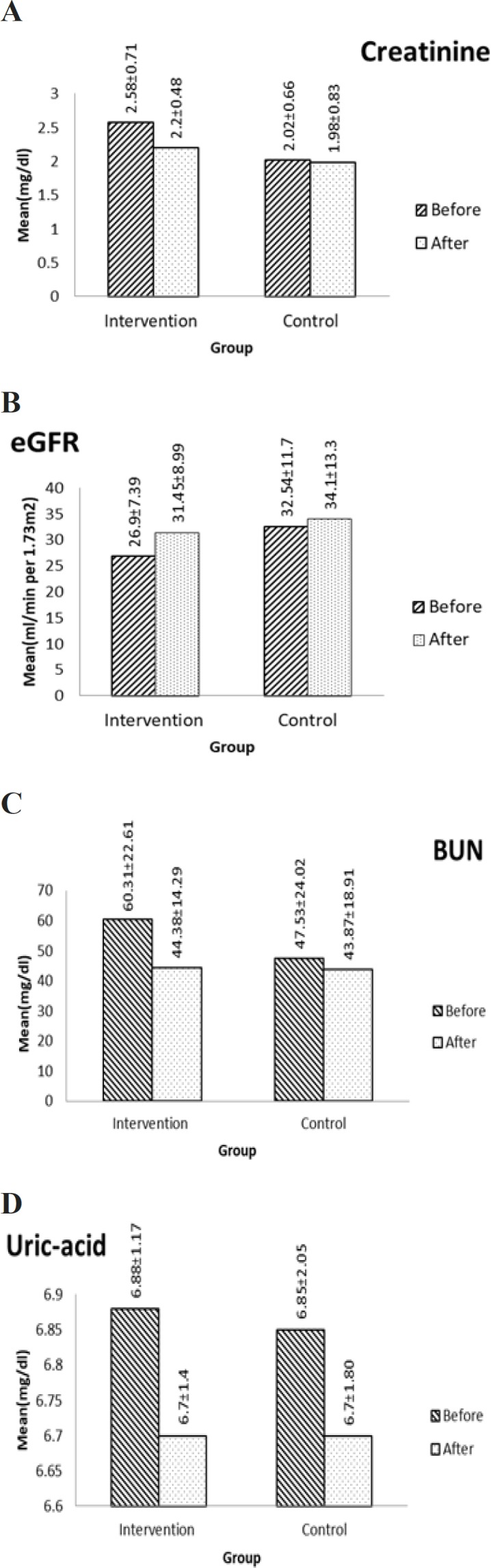
Before and after treatment values of renal function test including A: creatinine; B: eGFR; C: BUN and D: uric-acid in intervention (n=22) and control (n=22) groups; values are presented as mean±SD; BUN: Blood urea nitrogen

Serum creatinine and eGFR as well as other laboratory markers were investigated three months after the onset of the study. During the three months of treatment, eGFR was elevated significantly in the intervention group (p=0.001) and insignificantly in the control group (p=0.24). Differences in changes of GFR between the two groups were significant (intervention and control groups: 5.86±6.7 and 1.54±5.7, respectively; p=0.038). The treatment protocol of the study significantly reduced serum creatinine (p=0.01) and BUN (p=0.0001) in the intervention group. On the other hand, serum creatinine (p=0.75) and BUN (0.422) did not change significantly in the control group. Differences in variations of the serum creatinine (p=0.01) and Bun (p=0.008) between the two groups were significant. The combination therapy of camel milk and Tarangabin reduced uric acid more considerably than conventional therapy alone, though it was not significant (p=0.86) (Figure 2). 

Further, the differences in creatinine (p=0.82), BUN (p=0.95), and eGFR (p=0.65) were not significant between diabetic and hypertension patients in the intervention group (Table 2).

**Effect of combination therapy of camel milk and Tarangabin on glycemic control and BP**

**Figure 3:**
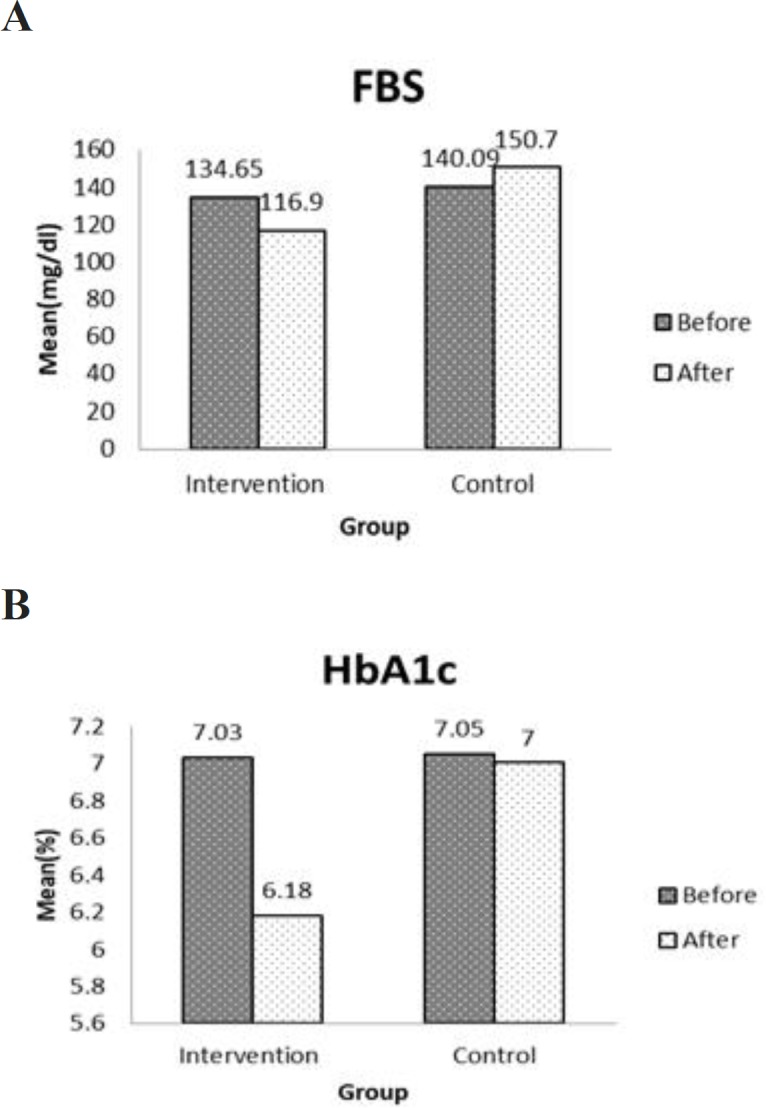
Before and after treatment values of A: FBS and B: HbA1c in the intervention (n=22) and control (n=22) groups. FBS: Fasting blood sugar; HbA1c: Hemoglobin A1C

FBS and HbA1c declined insignificantly at the end of the study in the intervention group (p=0.27 and p=0.14, respectively), while the mean of FBS increased (p=0.29) and HbA1c decreased (p=0.93) in the control group (Figure 3). The mean levels of FBS and HbA1c in diabetic participants in the intervention group diminished (168±46 to 133±48 mg/dl; p=0.344 and 8.0±0.68 to 7.8±1.17%; p=0.689, respectively). 

The effect of treatment protocol on SBP in the intervention [before and after, Median: 140 (130-150) and 130 (120-140); p=0.0001] and control groups [before and after, Median: 140 (110-190) and 137.5 (110-165); p=0.004) was significant. DBP dropped significantly in the intervention group [before and after, Median: 90 (80-100) and 80 (70-90); p=0.0001] and insignificantly in the control group [before and after, Median: 90 (80-100) and 90 (70-110); p=0.051].

**Table 2:** Comparison of renal function markers between subgroups based on the underling disease of CKD in the intervention group

	**Hypertension (n=12)**		**Diabetes (n=10)**		P value*
	**Before**	**After**	**Before**	**After**	
**BUN (** **mg/dl** **)**	62.7±27.4	44.2±12.9	57.4±16.0	44.6±16.5	0.95
**Serum Creatinine ** **(** **mg/dl** **)**	2.4±.7	2.1±.5	2.8±.7	2.2±.5	0.82
**GFR ** **(ml/min per 1.73 m2** **)**	26.9±7.1	30.4±8.9	25.2±6.1	32.7±9.4	0.56

*Data are presented as Mean±SD and analyzed by T test GFR: Glomerular filtration rate; BUN: Blood urea nitrogen

**Effect of combination therapy of camel milk and Tarangabin on lipid profile and other biochemical factors**

No significant changes in Ca, P, Na, and Hb levels, were observed during the study in the two groups. Levels of K dropped significantly (p=0.006) in the intervention group while no significant change was observed in the control group (p=0.427).

The lipid profile improved at the end of the study in all participants. Among the lipid profile variables, only triglyceride (TG) was significantly decreased in the intervention group (p=0.02) while changes in other variables were not significant (Table 3).

**Table 3:** Changes in biochemical factors after treatment in the study groups

laboratory characteristics*	**Intervention group**		**Control group**	
	**Before** **After**	**P value**	**Before** **After**	**P value**
Hb (mg/dl)	12.01±1.53	0.085	12.97±1.80	0.906
	12.48±1.3		13.01±1.86	
Na (mEq/L)	139.86±3.32	0.15	143.5±9.10	0.1
	138.8±3.2		139.1±4.7	
K (mEq/L)	4.43±0.66	0.006	4.54±0.95	0.427
	4.31±0.7		4.43±0.67	
Ca (mg/dl)	9.38±0.63	0.82	9.2±0.90	0.77
	9.4±0.4		9.3±0.57	
P (mg/dl)	4.10±0.68	0.96	4.43±1.43	0.67
	4.1±0.76		3.9±0.66	
TG (mg/dl)	182.95±95.19	0.02	144.72±69.98	0.96
	151.1±64.7		145.2±63.10	
Chol (mg/dl)	169.86±46.76	0.59	173.09±39.21	0.05
	165.4±32.3		157.5±36.10	
HDL (mg/dl)	39.86±10.83	0.21	48.27±15.83	0.05
	42.6±10.8		44.8±15.30	
LDL (mg/dl)	100.86±47.42	0.34	97.95±38.66	0.05
	92.7±28.9		82.5±25.20	

*Data are presented as Mean±SD and analyzed by T test or Wilcoxon signed-rank test, where appropriate; n=22 in each group; Hb: Hemoglobin; Na: Sodium; K: Potassium; Ca: Calcium; P: Phosphorus; TG: Triglyceride; Chol: Cholesterol; HDL: High-density lipoprotein; and LDL: Low-density lipoprotein

 The classification of patients based on CKD stages showed that 7 (31.8%) patients were at stage 3 of CKD (moderate decrease in GFR), 15 (68.2%) patients were at stage 4 (severe decline in GFR) in the intervention group, while 14 (63.6%) patients were at stage 3 and 8 (36.4%) were at stage 4 in the control group. At the end of the study, 5 patients transitioned from CKD stage 4 to CKD stage 3 in the intervention group. However, only 1 patient developed a change in the stage of the disease in the control group. 

 The treatment protocol in the intervention group did not have adverse effects. Only one patient reported abdominal pain in the intervention group and was excluded.

## Discussion

To the best of our knowledge, this study is the first clinical trial to examine the effect of combination therapy of camel milk with Tarangabin syrup plus conventional treatments in comparison with conventional treatments on CKD. The results of the study indicated that 400 cc of camel milk and 10 cc of 40% Tarangabin syrup daily given in two divided doses for 3 months along conventional treatments, was able to reduce serum creatinine, BUN, TG, and K, and DBP, and improve eGFR significantly. 

Several studies were conducted on the effect of camel milk on blood glucose control. For example, camel milk intake was associated with a decline in blood glucose levels in streptozotocin-induced diabetic rats (Agrawal et al., 2004; Khan et al., 2013). Also, Agrawal et al. showed that daily consumption of 500 ml of camel milk lowered the blood glucose in patients with type 1 diabetes (Agrawal, 2013 ▶; Agrawal, 2011a ▶). However, Ejtahed et al. (2015) ▶ observed that camel milk consumption did not show a significant effect on blood glucose in type 2 diabetic patients. Camel milk reduced HbA1c more than cow milk did (Agrawal et al., 2011b ▶), though in some studies this decrease was not significant (Mirmiran et al., 2017 ▶; Agarwal et al., 2003 ▶; El-Sayed, 2011 ▶). The results of this research indicated that reductions of blood glucose level and HbA1c in diabetic patients in the intervention group were not statistically significant. This was probably due to the fact that our diabetic participants had type 2 diabetes and camel milk might be more effective in insulin-dependent diabetic patients as it contains more insulin and insulin-like substances. 

Possibly, anti-diabetic properties of camel milk are due to existence of high concentrations of insulin and insulin-like substances. The camel milk insulin, due to the high buffering capacity of the camel milk, does not turn into clot in the acidic stomach. Its insulin also encapsulated in the form of lipid nanoparticles to protect it against proteolysis and facilitating its entry into the bloodstream. There are insulin-like small molecules in camel milk which bind to the insulin receptor and mimic its function (Malik et al., 2012 ▶). Further, the immunomodulatory properties of this milk on beta cells function is one its anti-diabetic mechanisms given its immunoglobulins of small size and weight (Kalla et al., 2017 ▶). 

 The effect of camel milk on kidney function parameters in *in vivo* models has also been proven (Hamad et al., 2011 ▶; Khan et al., 2013 ▶). In this regard, daily feeding of diabetic rats with camel milk significantly reduced creatinine (Khan et al., 2013 ▶; Korish et al., 2015 ▶) and serum urea. It also had a significant effect on renal function restoration and urine volume, and improved proteinuria (Korish et al., 2015 ▶). Similarly, Agrawal and Mohammed showed the role of camel milk in reducing proteinuria in diabetic nephropathy patients (Agrawal et al., 2009 ▶; Mohamad et al., 2009 ▶). 

 The histological changes caused by diabetic nephropathy including glomerular and tubular hypertrophy, include increased basement membrane thickness, tubulointerstitial fibrosis, arteriosclerosis, and diffuse mesangial matrix expansion (Ashraf et al., 2013 ▶). These histological changes were significantly improved by camel milk. Meanwhile, camel milk reduces insulin resistance, which inhibits progression of microvascular changes in diabetes. Recent reports support the effects of glucose-lowering agents on angiotensin II and advanced glycation end products (AGEs) reductions. Angiotensin II and AGEs stimulate production of Smad1 and collagen type IV (Col4). Camel milk can reduce Smad1 and Co14 production due to its antioxidant effects (Korish et al., 2015 ▶). Camel milk has also ACE inhibitory (ACE-I) effects. This milk’s whole casein and beta casein exhibit powerful ACE-I effects followed by hydrolysis of pepsins and tri-protease (Salami et al., 2011 ▶). 

Although the underlying causes of nephropathy are different, the events that contribute to the progression of the disease are similar. Inflammation and cytokine imbalance in all cases of CKD are present regardless of its initial cause, since any chemical or physical damage to the kidney cells activates inflammatory and fibrotic responses which ultimately lead to fibrosis and loss of nephrons and scars. Finally, fibrosis as well as mesangial and vascular contraction contributes to tubular degeneration, scarring and reduced GFR. Although the current treatment of CKD is based on renin–angiotensin inhibition, the anti-inflammatory and anti-fibrotic drugs will be more considered in the future (López-Novoa et al., 2010 ▶). Accordingly, it seems that camel milk, due to anti-inflammatory and anti-oxidant properties (Korish, 2014 ▶; Salami et al., 2011 ▶), is beneficial for reducing inflammation in CKD regardless of underlying causes. In the present study, there was no significant difference between diabetic and hypertension patients in intervention group in renal function tests data (Table 2).

Also, the immunostimulatory properties of Tarangabin syrup prescribed to the patients in this research, may contribute to the reduction of inflammation. The immunostimulatory properties of the total aqueous fraction of Tarangabin are attributed to its polysaccharide content (Hamedi et al., 2015 ▶). 

 According to TPM scholars, Tarangabin is laxative. The relationship between constipation and CKD was evaluated in a cohort study by Sumida et al. (2017) ▶. According to them, constipation and its severity are associated with increased incidence of CKD, ESRD, with continuous eGFR decline, independent of recognized risk factors. One of the reasons describing why constipation may be a risk factor for the progress of CKD, is altered gut microbiota by constipation (Sumida et al., 2017 ▶). Also, gastrointestinal motility and gut environment are interconnected (Quigley, 2011 ▶).

 Combined treatment of camel milk and Tarangabin with glucose regulating, ACE-I, anti-oxidant, anti-inflammatory, laxative, and immunostimulatory properties, seems to help to improve kidney function in CKD patients through preventing tissue damage and fibrosis.

The limitation of this study was the duration of the intervention. A longer study should be performed with a larger sample size. The patients should also be followed up after the intervention for 3 to 6 months. Further, the study groups can be defined based on underlying diseases and the effect of camel milk on CKD should be measured based on the underlying disease in future studies. It is recommended that in subsequent studies, the effect of camel milk be also evaluated on proteinuria. 

The results of this study suggest that camel milk with Tarangabin may contribute to a significant improvement in eGFR, serum creatinine, BUN and TG, k, DBP when administered along with common drugs in patients with CKD. It seems that the therapeutic protocol of this study can improve renal function in these patients; however, further studies are required to understand this effect precisely. This study could open a window for further researches. 
